# Supplementary material for: Multi‐dataset identification of innovative feature genes and molecular mechanisms in keratoconus
Source: J Cell Mol Med. 2024 Sep 19;28(18):e70079. doi: 10.1111/jcmm.70079 (PMC11412914; doi:10.1111/jcmm.70079)
Supplement: Supplementary file 2 — Material S2. [file JCMM-28-e70079-s002.zip › 7.GSEA analysis/6.SNORA74B/KEGG_REGULATION_OF_ACTIN_CYTOSKELETON.html]

Details for gene set KEGG\_REGULATION\_OF\_ACTIN\_CYTOSKELETON[GSEA]

|  || Dataset | SNORA74B.group.cls#h\_versus\_l |
| Phenotype | group.cls#h\_versus\_l |
| Upregulated in class | l |
| GeneSet | KEGG\_REGULATION\_OF\_ACTIN\_CYTOSKELETON |
| Enrichment Score (ES) | -0.31963757 |
| Normalized Enrichment Score (NES) | -1.3595723 |
| Nominal p-value | 0.051823415 |
| FDR q-value | 0.32481602 |
| FWER p-Value | 0.925 |
Table: GSEA Results Summary

  

Fig 1: Enrichment plot: KEGG\_REGULATION\_OF\_ACTIN\_CYTOSKELETON      
 Profile of the Running ES Score & Positions of GeneSet Members on the Rank Ordered List

  

| SYMBOL | TITLE | RANK IN GENE LIST | RANK METRIC SCORE | RUNNING ES | CORE ENRICHMENT || 1 | PIK3R5 | na | 170 | 0.800 | 0.0219 | No |
| 2 | FN1 | na | 587 | 0.575 | 0.0291 | No |
| 3 | ARHGEF6 | na | 1322 | 0.436 | 0.0225 | No |
| 4 | INSRR | na | 1547 | 0.412 | 0.0298 | No |
| 5 | RAC3 | na | 1795 | 0.390 | 0.0357 | No |
| 6 | FGF18 | na | 1820 | 0.388 | 0.0481 | No |
| 7 | BDKRB2 | na | 2580 | 0.327 | 0.0370 | No |
| 8 | FGF8 | na | 2974 | 0.302 | 0.0358 | No |
| 9 | FGF5 | na | 3327 | 0.285 | 0.0351 | No |
| 10 | FGF17 | na | 3644 | 0.272 | 0.0351 | No |
| 11 | TMSB4Y | na | 3920 | 0.262 | 0.0360 | No |
| 12 | PDGFRA | na | 4146 | 0.255 | 0.0380 | No |
| 13 | CYFIP2 | na | 4506 | 0.242 | 0.0357 | No |
| 14 | BCAR1 | na | 5506 | 0.208 | 0.0138 | No |
| 15 | FGF16 | na | 5815 | 0.198 | 0.0115 | No |
| 16 | SOS1 | na | 5971 | 0.193 | 0.0135 | No |
| 17 | MYL5 | na | 6916 | 0.165 | -0.0084 | No |
| 18 | BAIAP2 | na | 7054 | 0.161 | -0.0069 | No |
| 19 | ITGA4 | na | 7435 | 0.150 | -0.0129 | No |
| 20 | FGF3 | na | 7481 | 0.149 | -0.0092 | No |
| 21 | ITGB7 | na | 7862 | 0.139 | -0.0155 | No |
| 22 | ITGAL | na | 7931 | 0.138 | -0.0129 | No |
| 23 | BRAF | na | 8089 | 0.134 | -0.0130 | No |
| 24 | PIK3R1 | na | 8116 | 0.133 | -0.0093 | No |
| 25 | ARPC4 | na | 8193 | 0.131 | -0.0070 | No |
| 26 | CHRM5 | na | 8349 | 0.128 | -0.0072 | No |
| 27 | ROCK1 | na | 8851 | 0.117 | -0.0178 | No |
| 28 | FGFR4 | na | 9201 | 0.109 | -0.0243 | No |
| 29 | ITGA9 | na | 9643 | 0.100 | -0.0337 | No |
| 30 | ITGB6 | na | 9755 | 0.098 | -0.0337 | No |
| 31 | KRAS | na | 9766 | 0.097 | -0.0307 | No |
| 32 | IQGAP3 | na | 9782 | 0.097 | -0.0279 | No |
| 33 | PDGFA | na | 9861 | 0.095 | -0.0269 | No |
| 34 | MYH14 | na | 9976 | 0.093 | -0.0271 | No |
| 35 | VAV2 | na | 10064 | 0.091 | -0.0266 | No |
| 36 | GSN | na | 10144 | 0.090 | -0.0258 | No |
| 37 | LIMK1 | na | 10417 | 0.085 | -0.0309 | No |
| 38 | PIKFYVE | na | 10605 | 0.081 | -0.0336 | No |
| 39 | TMSB4X | na | 10678 | 0.080 | -0.0330 | No |
| 40 | FGF20 | na | 10959 | 0.076 | -0.0385 | No |
| 41 | DIAPH3 | na | 11092 | 0.073 | -0.0399 | No |
| 42 | FGFR3 | na | 11176 | 0.072 | -0.0399 | No |
| 43 | DIAPH2 | na | 11253 | 0.071 | -0.0397 | No |
| 44 | PIP4K2B | na | 11358 | 0.069 | -0.0404 | No |
| 45 | ACTB | na | 11421 | 0.068 | -0.0399 | No |
| 46 | FGF9 | na | 12036 | 0.059 | -0.0557 | No |
| 47 | SSH3 | na | 12048 | 0.059 | -0.0541 | No |
| 48 | F2R | na | 12299 | 0.056 | -0.0594 | No |
| 49 | PFN4 | na | 12353 | 0.055 | -0.0591 | No |
| 50 | ACTN4 | na | 12377 | 0.055 | -0.0579 | No |
| 51 | SSH2 | na | 12443 | 0.054 | -0.0580 | No |
| 52 | PIK3R2 | na | 12946 | 0.047 | -0.0710 | No |
| 53 | PIK3CA | na | 13079 | 0.046 | -0.0732 | No |
| 54 | SOS2 | na | 13685 | 0.039 | -0.0894 | No |
| 55 | FGF2 | na | 13739 | 0.039 | -0.0897 | No |
| 56 | FGF11 | na | 13944 | 0.036 | -0.0944 | No |
| 57 | CSK | na | 14025 | 0.035 | -0.0955 | No |
| 58 | IQGAP1 | na | 14103 | 0.035 | -0.0966 | No |
| 59 | ARHGEF4 | na | 14360 | 0.032 | -0.1029 | No |
| 60 | ITGAD | na | 14391 | 0.031 | -0.1027 | No |
| 61 | PDGFRB | na | 14565 | 0.029 | -0.1068 | No |
| 62 | FGF22 | na | 14606 | 0.029 | -0.1069 | No |
| 63 | MAP2K2 | na | 14904 | 0.025 | -0.1147 | No |
| 64 | ARPC3 | na | 14918 | 0.025 | -0.1142 | No |
| 65 | GIT1 | na | 14994 | 0.024 | -0.1156 | No |
| 66 | FGF13 | na | 15000 | 0.024 | -0.1149 | No |
| 67 | CHRM2 | na | 15078 | 0.024 | -0.1163 | No |
| 68 | ARPC5L | na | 15295 | 0.022 | -0.1219 | No |
| 69 | MYH9 | na | 15415 | 0.020 | -0.1246 | No |
| 70 | NCKAP1L | na | 15471 | 0.020 | -0.1256 | No |
| 71 | DIAPH1 | na | 15556 | 0.019 | -0.1274 | No |
| 72 | ABI2 | na | 15746 | 0.017 | -0.1323 | No |
| 73 | PIK3CB | na | 15769 | 0.017 | -0.1323 | No |
| 74 | MYL12B | na | 16040 | 0.014 | -0.1397 | No |
| 75 | VAV3 | na | 16097 | 0.014 | -0.1408 | No |
| 76 | TIAM1 | na | 16272 | 0.012 | -0.1455 | No |
| 77 | CD14 | na | 16315 | 0.011 | -0.1463 | No |
| 78 | ARHGAP35 | na | 16478 | 0.010 | -0.1507 | No |
| 79 | WAS | na | 16570 | 0.009 | -0.1530 | No |
| 80 | INS | na | 16698 | 0.007 | -0.1565 | No |
| 81 | MAPK3 | na | 16753 | 0.007 | -0.1578 | No |
| 82 | MOS | na | 16949 | 0.005 | -0.1633 | No |
| 83 | PAK4 | na | 17175 | 0.003 | -0.1697 | No |
| 84 | FGF10 | na | 17212 | 0.003 | -0.1706 | No |
| 85 | FGF19 | na | 17258 | 0.002 | -0.1719 | No |
| 86 | PPP1CB | na | 17283 | 0.002 | -0.1725 | No |
| 87 | EGF | na | 17351 | 0.001 | -0.1744 | No |
| 88 | ITGB2 | na | 17416 | 0.000 | -0.1763 | No |
| 89 | ITGB4 | na | 17477 | -0.000 | -0.1780 | No |
| 90 | IQGAP2 | na | 17497 | -0.001 | -0.1785 | No |
| 91 | WASF2 | na | 17499 | -0.001 | -0.1785 | No |
| 92 | ROCK2 | na | 17655 | -0.002 | -0.1830 | No |
| 93 | DOCK1 | na | 17724 | -0.003 | -0.1848 | No |
| 94 | WASL | na | 17732 | -0.003 | -0.1849 | No |
| 95 | ARPC2 | na | 17775 | -0.003 | -0.1860 | No |
| 96 | CFL1 | na | 17798 | -0.004 | -0.1866 | No |
| 97 | ARPC5 | na | 17932 | -0.005 | -0.1902 | No |
| 98 | NCKAP1 | na | 17980 | -0.006 | -0.1914 | No |
| 99 | ACTG1 | na | 18268 | -0.009 | -0.1994 | No |
| 100 | RHOA | na | 18290 | -0.009 | -0.1997 | No |
| 101 | ARPC1A | na | 18393 | -0.011 | -0.2023 | No |
| 102 | PPP1CA | na | 18424 | -0.011 | -0.2028 | No |
| 103 | PIP5K1C | na | 18480 | -0.012 | -0.2040 | No |
| 104 | MYL12A | na | 18525 | -0.012 | -0.2049 | No |
| 105 | CDC42 | na | 18770 | -0.015 | -0.2115 | No |
| 106 | PIK3CG | na | 18884 | -0.016 | -0.2142 | No |
| 107 | RAC2 | na | 18892 | -0.016 | -0.2139 | No |
| 108 | PAK6 | na | 18984 | -0.017 | -0.2159 | No |
| 109 | CYFIP1 | na | 19185 | -0.019 | -0.2211 | No |
| 110 | FGF14 | na | 19449 | -0.022 | -0.2280 | No |
| 111 | EGFR | na | 19596 | -0.024 | -0.2314 | No |
| 112 | HRAS | na | 19699 | -0.025 | -0.2335 | No |
| 113 | RAC1 | na | 19881 | -0.027 | -0.2378 | No |
| 114 | PIK3CD | na | 20354 | -0.032 | -0.2504 | No |
| 115 | FGF12 | na | 20421 | -0.033 | -0.2512 | No |
| 116 | FGF7 | na | 20472 | -0.034 | -0.2515 | No |
| 117 | MYLK | na | 20559 | -0.035 | -0.2529 | No |
| 118 | MAPK1 | na | 20606 | -0.035 | -0.2530 | No |
| 119 | F2 | na | 20714 | -0.037 | -0.2549 | No |
| 120 | RAF1 | na | 21074 | -0.041 | -0.2639 | No |
| 121 | PAK3 | na | 21207 | -0.042 | -0.2663 | No |
| 122 | PPP1CC | na | 21514 | -0.046 | -0.2736 | No |
| 123 | BRK1 | na | 21542 | -0.046 | -0.2729 | No |
| 124 | ARHGEF7 | na | 21618 | -0.047 | -0.2735 | No |
| 125 | PFN1 | na | 22005 | -0.052 | -0.2829 | No |
| 126 | ITGA2 | na | 22079 | -0.053 | -0.2832 | No |
| 127 | PPP1R12A | na | 22318 | -0.056 | -0.2883 | No |
| 128 | ITGA11 | na | 22351 | -0.056 | -0.2873 | No |
| 129 | WASF1 | na | 22378 | -0.057 | -0.2862 | No |
| 130 | VCL | na | 22408 | -0.057 | -0.2851 | No |
| 131 | APC2 | na | 22646 | -0.060 | -0.2900 | No |
| 132 | PTK2 | na | 22693 | -0.061 | -0.2892 | No |
| 133 | EZR | na | 23054 | -0.066 | -0.2975 | No |
| 134 | ITGA3 | na | 23349 | -0.071 | -0.3036 | No |
| 135 | SLC9A1 | na | 23712 | -0.076 | -0.3115 | No |
| 136 | MYH10 | na | 23780 | -0.077 | -0.3109 | No |
| 137 | ITGA2B | na | 23836 | -0.078 | -0.3098 | No |
| 138 | MYL2 | na | 23893 | -0.079 | -0.3088 | No |
| 139 | GNG12 | na | 23995 | -0.081 | -0.3090 | No |
| 140 | PFN2 | na | 24113 | -0.082 | -0.3097 | No |
| 141 | FGFR2 | na | 24244 | -0.085 | -0.3106 | No |
| 142 | LIMK2 | na | 24245 | -0.085 | -0.3077 | No |
| 143 | TIAM2 | na | 24277 | -0.085 | -0.3058 | No |
| 144 | ITGB5 | na | 24428 | -0.088 | -0.3072 | No |
| 145 | MYLK2 | na | 24506 | -0.089 | -0.3064 | No |
| 146 | RRAS | na | 24557 | -0.090 | -0.3048 | No |
| 147 | GNA12 | na | 24641 | -0.092 | -0.3041 | No |
| 148 | FGF6 | na | 24652 | -0.092 | -0.3013 | No |
| 149 | RDX | na | 24964 | -0.098 | -0.3071 | No |
| 150 | ITGA7 | na | 25192 | -0.102 | -0.3102 | No |
| 151 | FGF1 | na | 25243 | -0.103 | -0.3082 | No |
| 152 | ENAH | na | 25545 | -0.108 | -0.3133 | No |
| 153 | RRAS2 | na | 25730 | -0.112 | -0.3149 | No |
| 154 | PAK2 | na | 25754 | -0.112 | -0.3118 | No |
| 155 | PIP4K2C | na | 25967 | -0.116 | -0.3140 | No |
| 156 | PXN | na | 26060 | -0.118 | -0.3128 | No |
| 157 | BDKRB1 | na | 26112 | -0.119 | -0.3102 | No |
| 158 | ARHGEF12 | na | 26140 | -0.120 | -0.3070 | No |
| 159 | ARPC1B | na | 26427 | -0.126 | -0.3111 | No |
| 160 | ITGAV | na | 26724 | -0.133 | -0.3152 | Yes |
| 161 | CRK | na | 26740 | -0.134 | -0.3111 | Yes |
| 162 | MYLK3 | na | 26822 | -0.135 | -0.3089 | Yes |
| 163 | CHRM3 | na | 26903 | -0.138 | -0.3066 | Yes |
| 164 | PIP4K2A | na | 26936 | -0.138 | -0.3029 | Yes |
| 165 | SCIN | na | 27047 | -0.140 | -0.3014 | Yes |
| 166 | GNA13 | na | 27061 | -0.141 | -0.2971 | Yes |
| 167 | FGF21 | na | 27099 | -0.142 | -0.2934 | Yes |
| 168 | PFN3 | na | 27398 | -0.149 | -0.2970 | Yes |
| 169 | ITGB3 | na | 27418 | -0.149 | -0.2926 | Yes |
| 170 | ITGB1 | na | 27543 | -0.152 | -0.2910 | Yes |
| 171 | ITGAM | na | 27645 | -0.155 | -0.2888 | Yes |
| 172 | ARAF | na | 27916 | -0.162 | -0.2911 | Yes |
| 173 | ITGA6 | na | 27950 | -0.163 | -0.2866 | Yes |
| 174 | NRAS | na | 27969 | -0.164 | -0.2816 | Yes |
| 175 | ACTN1 | na | 28221 | -0.171 | -0.2832 | Yes |
| 176 | ITGAE | na | 28256 | -0.172 | -0.2784 | Yes |
| 177 | PAK1 | na | 28478 | -0.179 | -0.2788 | Yes |
| 178 | FGD1 | na | 28905 | -0.192 | -0.2847 | Yes |
| 179 | CRKL | na | 29271 | -0.203 | -0.2885 | Yes |
| 180 | PIK3R3 | na | 29453 | -0.208 | -0.2867 | Yes |
| 181 | ITGA10 | na | 29479 | -0.209 | -0.2805 | Yes |
| 182 | SSH1 | na | 29792 | -0.218 | -0.2822 | Yes |
| 183 | APC | na | 30020 | -0.226 | -0.2812 | Yes |
| 184 | PDGFB | na | 30041 | -0.227 | -0.2741 | Yes |
| 185 | FGF23 | na | 30156 | -0.231 | -0.2697 | Yes |
| 186 | CHRM1 | na | 30177 | -0.232 | -0.2625 | Yes |
| 187 | ACTN2 | na | 30345 | -0.239 | -0.2593 | Yes |
| 188 | MYL9 | na | 30587 | -0.249 | -0.2579 | Yes |
| 189 | MSN | na | 30763 | -0.256 | -0.2544 | Yes |
| 190 | ARHGEF1 | na | 31077 | -0.272 | -0.2543 | Yes |
| 191 | MAP2K1 | na | 31129 | -0.275 | -0.2466 | Yes |
| 192 | FGD3 | na | 32053 | -0.325 | -0.2624 | Yes |
| 193 | ITGB8 | na | 32130 | -0.330 | -0.2536 | Yes |
| 194 | CHRM4 | na | 32284 | -0.339 | -0.2466 | Yes |
| 195 | ITGAX | na | 32615 | -0.361 | -0.2441 | Yes |
| 196 | VAV1 | na | 32928 | -0.388 | -0.2401 | Yes |
| 197 | PIP5K1A | na | 33089 | -0.403 | -0.2312 | Yes |
| 198 | ITGA5 | na | 33190 | -0.413 | -0.2203 | Yes |
| 199 | ACTN3 | na | 33200 | -0.413 | -0.2067 | Yes |
| 200 | MYL7 | na | 33280 | -0.422 | -0.1948 | Yes |
| 201 | CFL2 | na | 33537 | -0.454 | -0.1870 | Yes |
| 202 | ITGA8 | na | 33651 | -0.470 | -0.1745 | Yes |
| 203 | ITGA1 | na | 33862 | -0.506 | -0.1636 | Yes |
| 204 | MYL10 | na | 34073 | -0.549 | -0.1513 | Yes |
| 205 | FGF4 | na | 34349 | -0.632 | -0.1381 | Yes |
| 206 | PDGFC | na | 34382 | -0.645 | -0.1174 | Yes |
| 207 | PDGFD | na | 34541 | -0.723 | -0.0977 | Yes |
| 208 | MRAS | na | 34571 | -0.753 | -0.0733 | Yes |
| 209 | FGFR1 | na | 34687 | -0.963 | -0.0443 | Yes |
| 210 | PIP5K1B | na | 34724 | -1.356 | 0.0001 | Yes |
Table: GSEA details [plain text format]

  

Fig 2: KEGG\_REGULATION\_OF\_ACTIN\_CYTOSKELETON      
 Blue-Pink O' Gram in the Space of the Analyzed GeneSet

  

Fig 3: KEGG\_REGULATION\_OF\_ACTIN\_CYTOSKELETON: Random ES distribution      
 Gene set null distribution of ES for **KEGG\_REGULATION\_OF\_ACTIN\_CYTOSKELETON**
